# Editorial: Women in integrative physiology: 2021

**DOI:** 10.3389/fphys.2022.1036614

**Published:** 2022-09-30

**Authors:** Gisela Helfer, Mahita Kadmiel, Preeti H. Jethwa

**Affiliations:** ^1^ School of Chemistry and Biosciences, Faculty of Life Sciences, University of Bradford, Bradford, United Kingdom; ^2^ Department of Biology, Allegheny College, Meadville, PA, United States; ^3^ School of Biosciences, Faculty of Science, University of Nottingham, Loughborough, United Kingdom

**Keywords:** physiology, fetal developement, neuroinflamamation, maternal stress, cardiac physiology

Women have been active in STEM subjects, including the field of physiology, since the early days, yet, less than 30% of researchers worldwide are women. On top of this, the contribution of women to the field often receives little coverage. Anecdotally, our students (and many researchers) often seem to find it difficult to name important female physiologists. Despite being under acknowledged, female physiologists have been crucial to scientific advances with 12 women so far having been awarded the Nobel prize in Physiology or Medicine. However, this is in stark contrast to 212 men!.

Encouragingly, the number of female researchers in physiology is increasing, thanks to proactive approaches such as the United Nations’ International Day of Women and Girls in Science, a day which celebrates outstanding achievements of female researchers and encourages more girls to study STEM subjects. At higher education level, charters like the Athena SWAN awards at universities in the United Kingdom, provide turning points and force the sector to review policies and practices to increase gender equality. Additionally, bespoke grants including the L’Oreal-UNESCO for Women in Science and renowned leadership development initiatives tailored to females, for example Aurora from Advanced HE and Sustain from the Academy of Medical Science provide inspiration and guidance and help advance the representation of women in science. Over in the United States, the National Science Foundation has initiatives such as the ADVANCE program to support gender diversity of faculty in STEM, with a special initiative for women with disabilities in STEM in academia.

But there is an urgent need to keep breaking any structural barriers and unfair processes that are still in place in the higher education sector. For example, gender bias, often unconscious, during interviews, salary negotiation, peer-review processes in publication and grant awards, promotions and short-term contracts are often difficult for women (and men) with caring responsibilities and the “leaky” pipeline in career progression means that fewer women make it to professor or senior management level. This is of course also true for physiology. Access to childcare at scientific meetings and conferences should continue to improve dramatically to facilitate participation of women scientists with young children who are mostly at the key juncture of transitioning from training to independent positions as scientists. To recruit and retain women in the field of physiology, it would help for the younger generation to see more women physiologists as role models such as educators in their classrooms, in leadership positions at their institutions, and speakers at conferences. Change can come faster also by women physiologists taking the stance to build camaraderie, to support each other and pay forward by creating opportunities for the next generation of women in physiology.

When Frontiers approached us with the idea to celebrate the work led and achieved by women in integrative physiology, we were delighted. The research topic Women in Integrative Physiology 2021 honors the research led and achieved by female researchers in this field and showcases their creativity, innovation and achievements.

Physiology is a diverse subject that deals with complex living organisms, from cells and molecules to organs and organ system levels. The diversity of Integrative Physiology is reflected by the articles submitted to this special issue. We have received four great articles covering neuroendocrinology, nutritional physiology, female reproductive biology and cardiac physiology, from early career to senior female researchers.

Yun et al. demonstrated a central effect of chemerin by manipulating chemerin signaling in the hypothalamus, a brain region associated with appetite regulation. Using pharmacological and genetic manipulation approaches, they found that the chemerin receptor CMKLR1 is functionally important for the central effects of chemerin on body weight regulation and neuroinflammation and implicate the chemerin-CMKLR1 axis in the regulation of cognition. While Alliband et al. are the first group, led by a female researcher, to have scoped the literature to show that vitamin D directly affects myogenesis by inhibiting cell proliferation and promoting differentiation, suggesting that lack of vitamin D during embryonic and fetal development could impact muscle development. Gotlieb et al. investigated the neuroendocrine mechanisms by which stress can lead to negative pregnancy outcomes and revealed that psychological stress during pregnancy altered circulating levels of glucocorticoids, and that those dams exhibited slower weight gain during gestation and were qualitatively more likely to experience delays in fetal development. The final article by Diaz-Canestro et al. investigated the contribution of blood volume and oxygen capacity on sex differences in cardiorespiratory fitness in healthy young individuals and concluded that normalisation of blood abolished sex differences in aerobic capacity.

We would like to thank all authors for their excellent contribution to the Research Topic Women in Integrative Physiology and the reviewers for their constructive feedback. The editorial staff have been incredibly supportive and assisted us during the process. Importantly, we want to thank them for the excellent idea to help promote research by women in integrative physiology. We hope that this Research Topic, even if only in a small way, will contribute to more visibility of the amazing research that women are contributing to the field. Physiology is an exciting subject and we are positive that by working together we will break the bias and encourage more female students to remain in the field and take up the opportunity to become role models themselves for the next generation of female physiologists.

